# Clinical benefit of metaphase I oocytes

**DOI:** 10.1186/1477-7827-3-71

**Published:** 2005-12-15

**Authors:** Leen Vanhoutte, Petra De Sutter, Josiane Van der Elst, Marc Dhont

**Affiliations:** 1 Infertility Centre, Ghent University Hospital, De Pintelaan 185, 9000 Ghent, Belgium

## Abstract

**Background:**

We studied the benefit of using in vitro matured metaphase I (MI) oocytes for ICSI in patients with a maximum of 6 mature metaphase II (MII) oocytes at retrieval.

**Methods:**

In 2004, 187 ICSI cycles were selected in which maximum 6 MII oocytes and at least one MI oocyte were retrieved. MI oocytes were put in culture to mature until the moment of ICSI, which was performed between 2 to 11 hours after oocyte retrieval (day 0). In exceptional cases, when the patient did not have any mature oocyte at the scheduled time of ICSI, MI oocytes were left to mature overnight and were injected between 19 to 26 hours after retrieval (day 1). Embryos from MI oocytes were chosen for transfer only when no other good quality embryos from MII oocytes were available. Outcome parameters were time period of in vitro maturation (IVM), IVM and fertilization rates, embryo development, clinical pregnancy rates, implantation rates and total MI oocyte utilization rate.

**Results:**

The overall IVM rate was 43%. IVM oocytes had lower fertilization rates compared to in vivo matured sibling oocytes (52% versus 68%, P < 0.05). The proportion of poor quality embryos was significantly higher in IVM derived oocytes. One pregnancy and live birth was obtained out of 13 transfers of embryos exclusively derived from IVM oocytes. This baby originated from an oocyte that was injected after 22 hrs of IVM.

**Conclusion:**

Fertilization of in vitro matured MI oocytes can result in normal embryos and pregnancy, making IVM worthwhile, particularly when few MII oocytes are obtained at retrieval.

## Background

A proportion of human oocytes collected during an IVF or ICSI procedure remains meiotically immature at the germinal vesicle (GV) or metaphase I (MI) stage. Several publications have shown that this proportion fluctuates around 15 to 20% [1,2]. It is not exactly known why some of the oocytes are unresponsive to the maturation trigger in vivo. Different explanations are possible. When ovarian hyperstimulation is started, follicles may be at different stages of development, producing oocytes of varying degrees of maturity. Since follicles are aspirated prior to rupture, the collected oocytes come from a heterogeneous pool of follicles, including luteinizing as well as degenerating follicles [3]. It is also possible that smaller antral follicles are aspirated during oocyte retrieval, which can result in the collection of immature oocytes [4,5]. Eventually, the proportion of immature oocytes can be dependent on patients' characteristics (such as cause of infertility, age, ovarian reserve) and the stimulation protocol used.

Immature oocytes from superovulated cycles can undergo the final stages of meiotic maturation spontaneously in vitro. MI oocytes have already undergone the process of germinal vesicle breakdown (GVBD) and may progress to the metaphase II (MII) stage within a few hours of in vitro culture. This allows them to be injected by ICSI at the same time as their sibling mature MII oocytes. The clinical use of MI oocytes from stimulated cycles has been studied by several research groups. It has been reported that these in vitro matured oocytes yield lower fertilization rates [6-8], abnormal embryonic development [7-9] and lower implantation rates [6] than in vivo matured oocytes. Development to term is limited to rare cases [6,8,9]. As a consequence, immature oocytes from stimulated cycles are generally considered to be a side-product and only in vivo matured MII oocytes are used for ICSI. Nevertheless, for patients in whom a low number of MII oocytes are retrieved, the use of in vitro matured MI oocytes may be worthwhile in order to increase the number of injectable oocytes at the time of ICSI.

We therefore designed the present study to determine if, indeed, in vitro matured MI oocytes could have a clinical application in our IVF-program in a selected population of patients with a low number of MII oocytes at retrieval. This was analyzed within the framework of a daily laboratory practice, without changing the routine of ovarian stimulation, oocyte retrieval, ICSI procedure, embryo culture or transfer.

## Methods

### Patient selection

The study included all ICSI cycles over a one year period (2004) in which two inclusion criteria were fulfilled: 1) a maximum of 6 mature (MII) oocytes and 2) at least one MI oocyte present at retrieval.

Our ICSI program has been approved as infertility treatment by the Ghent University Hospital Ethical Committee.

### Ovarian stimulation, IVM and oocyte handling for ICSI

All patients underwent controlled ovarian stimulation after cycle synchronization with a standard contraceptive pill for 2–6 weeks. A short gonadotrophin-releasing hormone (GnRH) agonist protocol was used, consisting of 0.1 mg of triptorelin (Decapeptyl, Ipsen, France) from day 5 onwards after discontinuation of the oral contraceptive. This was followed by human menopausal gonadotrophin (hMG; Menopur, Ferring, Germany) or follicle stimulating hormone (FSH; either Gonal-F, Serono, Switzerland or Puregon, Organon, The Netherlands) from day 7 after discontinuation of the pill onwards. The starting dose was usually 150 IU, but this dose was adjusted after 7 days of hMG or FSH administration, according to the individual response of the patient. Known poor responders were started on 300 IU of hMG or FSH daily. The follicular phase was monitored by means of transvaginal ultrasound scanning of the ovaries and serum estradiol measurement if judged necessary. An injection of 5,000 or 10,000 IU human chorionic gonadotrophin (hCG; Pregnyl, Organon, The Netherlands) was administered when half of all mature follicles had reached a mean diameter of at least 20 mm, measured in two planes. Oocyte retrieval was scheduled 34 to 36 hrs after hCG administration.

Oocytes were denuded enzymatically by a brief exposure of the cumulus-oocyte complexes to 80 IU/ml hyaluronidase (Type VIII; Sigma Chemical Co., Bornem, Belgium), followed by mechanical denudation approximately 1 to 2 hrs after oocyte collection. The nuclear status of denuded oocytes was subsequently recorded. GV oocytes were not considered for ICSI. MI oocytes were defined as those oocytes in which no GV and no first polar body were visible. These oocytes were put in culture to mature. The culture medium for in vitro maturation (IVM) was either Sydney IVF Fertilization Medium (Cook, Ltd., Limerick, Ireland) or Early Cleavage Medium (Irvine Scientific, Brussels, Belgium).

MI oocytes were left to mature until the time when ICSI for the particular patient was carried out. This was in a time-frame of 2 to 11 hrs after oocyte retrieval (day 0). In exceptional cases, when the patient did not have any mature oocyte at the scheduled time of ICSI, MI oocytes were left to mature overnight and, when matured, were injected between 19 to 26 hrs after oocyte retrieval (day 1).

In vivo and/or in vitro matured oocytes were injected with patient's sperm. Sperm preparation for ICSI and details for the microinjection procedure have been described elsewhere [10]. After injection, oocytes were cultured in either Sydney IVF Cleavage Medium (Cook) or Early Cleavage Medium.

### Embryo evaluation and transfer

Assessment of fertilization took place between 16 to 20 hrs after ICSI. Embryos were evaluated based on the number of blastomeres and the degree of fragmentation. Embryos with less than 10% anucleated fragments were classified as 'excellent'. Embryos with either 10–20% or >20% anucleated fragments were classified as 'good' and 'poor' quality embryos, respectively. Embryos with at least one blastomere with more than one nucleus were classified as multinucleated embryos and were considered as 'poor' quality embryos as well, regardless of the degree of fragmentation.

Transfer of embryos was carried out on day 2 or day 3. Embryos from in vitro matured MI oocytes were chosen for transfer only when no other good quality embryos from MII oocytes were available. The number of embryos transferred was limited by Belgian law based on female age, cycle number and embryo quality [11].

Pregnancy was diagnosed by the detection of a positive serum hCG at least 14 days after embryo transfer, followed by a rise in hCG levels. All patients received a transvaginal ultrasound scan between 6 and 7 weeks of pregnancy to differentiate between biochemical and clinical (presence of an intra-uterine gestational sac with fetal heart beat) pregnancies and to diagnose ectopic implantations. All pregnancies were monitored further by transvaginal ultrasound until 12 weeks of amenorrhoea.

### Statistical analysis

For comparison among groups, results were analyzed with Chi-square and Fisher's exact test when appropriate. When the P value was <0.05, the difference was considered significant.

## Results

### Patient and cycle characteristics

In the year 2004, 180 patients underwent 187 ICSI cycles in which maximum 6 MII oocytes and at least one MI oocyte were retrieved. This is 12.7% of the total number of ICSI cycles performed in our infertility center during the same year. The mean age of the patients was 35.9 ± 4.67 years (range 25–48).

A total of 1208 oocytes were collected. Three hundred of these oocytes were at the MI stage at the moment of oocyte denudation (24.8%; mean of 1.6 MI oocytes/cycle; range 1–6). Hundred thirty-two oocytes were at the GV stage (10.9%) and 80 oocytes (6.6%) were degenerated or damaged at the moment of denudation.

### Comparison between in vitro matured MI oocytes and in vivo matured oocytes

Overall, 43% (129/300) of MI oocytes matured to the MII stage. Maturation and injection of at least one MI oocyte was achieved in approximately half of the ICSI cycles (55%; n = 102). In vivo matured sibling MII oocytes, retrieved in the same treatment cycles, were injected in parallel and served as the control group. ICSI was performed with fresh ejaculate in 81.4% of the cycles and with frozen ejaculate in 9.8% of the cycles. Frozen epididymal or testicular spermatozoa were used in respectively 1.0% and 7.8% of the cycles.

The results of fertilization and embryo development in the two groups of oocytes (IVM + control) are presented in Table 1. The fertilization rate of matured MI oocytes was significantly lower compared to the fertilization rate of in vivo matured oocytes (52% versus 68%, P < 0.05). Also the embryo quality was different between the two groups. In the in vitro matured group, significantly less embryos of excellent quality and more embryos of poor quality were obtained compared to the in vivo matured group. This was observed on day 2 (p < 0.05) as well as on day 3 (p < 0.05).

**Table 1:** Comparison of fertilization and embryonic development between in vitro and in vivo matured sibling oocytes

	**In vitro matured MI oocytes**	**In vivo matured sibling oocytes (control)**
**Total N° of oocytes injected**	129	339
**N° (%) of normal fertilized oocytes**^**a**^	67 (52)*	229 (68)
**Embryo development on day 2 (%)**^**b**^		
**Excellent**	24*	41
**Good**	27	32
**Poor**	49*	27
**Embryo development on day 3 (%)**^**b**^		
**Excellent**	22*	38
**Good**	28	32
**Poor**	50*	30

* Statistically different between columns (p < 0.05).

^a ^Normal fertilized oocytes were defined as oocytes containing 2 pronuclei en 2 polar bodies.

^b ^The proportions of excellent, good and poor embryos are expressed per total number of embryos obtained on day 2 or day 3.

### Comparison between different time periods of IVM

A second evaluation of the results was done by splitting up the period of IVM, between collection of the oocytes and the time of ICSI, in different time intervals (Table 2).

**Table 2:** Comparison of maturation rates, fertilization rates and embryo development between different time periods of IVM

**Time interval of IVM**	**N° of MI oocytes**	**N° (%) of in vitro matured oocytes**	**N° (%) of fertilized oocytes**	**N° (%) of excellent + good quality embryos on Day 2 **^**(c)**^	**N° (%) of excellent + good quality embryos on Day 3**
**2 to 4 hrs**	147	60 (41)	26 (43)^a^	15 (58)	14 (61)
**>4 to 7 hrs**	136	57 (42)	31 (54)^a^	15 (48)	9 (38)
**>7 to 11 hrs**	9	6 (67)	6 (100)^b^	3 (50)	2 (40)
**19 to 26 hrs**	8	6 (75)	4 (67)^ab^	1 (25)	2 (100)

^a,b ^Different letters indicate significant differences within columns (p < 0.05).

^c ^Thirteen embryos were transferred on day 2.

A number of 147 oocytes, coming from 100 ICSI cycles, were evaluated for maturity within 2–4 hrs of IVM culture, 136 oocytes (77 ICSI cycles) within >4–7 hrs of culture, 9 oocytes (5 ICSI cycles) within >7–11 hrs of culture, and 8 oocytes (5 ICSI cycles) were left to mature overnight and were evaluated the day after retrieval, between 19–26 hrs of culture. A time-dependent increase in the progression to maturation was noted, ranging from 41% mature oocytes after 2–4 hrs of IVM to 75% after 19–26 hrs of IVM, but this trend was not statistically different. The fertilization rate in the group of >7–11 hrs of IVM (100%) was significantly higher compared to 2–4 hrs of IVM (43%) and >4–7 hrs of IVM (54%) (p < 0.05), but not to 19–26 hrs of IVM (67%). There was no difference between the number of excellent and good quality embryos in the different IVM time interval groups.

### Embryo transfer, clinical pregnancy and implantation rates

The most important parameter to evaluate whether the use of in vitro matured MI oocytes has a clinical benefit is the pregnancy outcome. Table 3 represents the results of embryo transfer, clinical pregnancy and implantation rates. A distinction was made between cycles in which at least one MI oocyte was matured in vitro (n = 102) and ICSI cycles with no matured MI oocytes (n = 85). The group of cycles with matured MI oocytes was further split-up in 3 groups: 1) transfers involving exclusively embryos derived from in vitro matured MI oocytes, 2) mixed transfers and 3) transfers involving exclusively embryos derived from in vivo matured MII oocytes. A double embryo transfer involving embryos exclusively derived from MI oocytes resulted in a singleton pregnancy and the birth of a healthy baby girl. This baby originated from an oocyte that was injected after 22 hrs of IVM. There was no statistical difference between clinical pregnancy rates and embryo implantation rates in the three groups. However, when the numbers of exclusively MI and mixed transfers were pooled and compared to exclusively MII transfers, the clinical pregnancy rates (8.8% versus 27.0%) and embryo implantation rates (5.6% versus 15.1%) were significantly lower in the former group (p < 0.05).

**Table 3:** Clinical pregnancy and implantation rates in cycles with maximum 6 MII oocytes

	**cycles with at least one MI oocyte matured (n = 102)**			**cycles with no MI oocyte matured (n = 85)**	**Total (n = 187)**^**a**^
			
	**Exclusively embryos derived from MI oocytes**	**Mixed transfers**	**Exclusively embryos derived from sibling MII oocytes**		
**N° of transfers**	13	21	63	81	178
**N° of embryos transferred**	17	54	119	141	331
**N° (%) of clinical pregnancies per embryo transfer**	1 (7.7)	2 (9.5)	17 (27.0)	15 (18.5)	35 (19.7)
**N° (%) of implanted embryos**	1 (5.9)	3 (5.6)^b^	18 (15.1)^b^	15 (10.6)	37 (11.2)

No statistical difference between columns.

^a ^In 9 ICSI cycles, no embryo transfer was done.

^b ^A twin pregnancy was obtained in these groups.

### Total MI oocyte utilization rate

Forty-two embryos derived from normally fertilized in vitro matured oocytes were used for transfer and 9 embryos were cryopreserved. This means that 51 out of 67 embryos originating from MI oocytes were used. The total MI oocyte utilization rate (= percentage of embryos transferred and frozen per fertilized oocyte) was 76%.

## Discussion

The present study aimed to analyze, for the first time, the clinical benefit of MI oocytes in a selected group of patients with a low number of mature oocytes at retrieval. To achieve this goal, we selected ICSI cycles in which a maximum of 6 mature oocytes and at least one MI oocyte were obtained at oocyte retrieval. The results show that fertilization rate and developmental capacity of the embryos was significantly reduced in IVM oocytes compared with control sibling oocytes. One live birth obtained after transfer of embryos exclusively derived from IVM oocytes illustrates that the use of MI oocytes is not of major issue in IVF programs, but may be an option for patients with low numbers of MII oocytes.

A low number of oocytes at retrieval might be a result of low ovarian response to gonadotrophin stimulation. Low response to stimulation occurs in approximately 10% of the ART population [12]. There is no universally accepted definition for low response. One of the criteria is the number of oocytes retrieved. Faber et al. [13] used an oocyte retrieval rate of ≤ 4 mature oocytes as cut-off limit, while De Sutter et al. [14] and Moreno et al. [15] categorized < 5 and ≤ 6 oocytes retrieved, respectively, as low responding patients, without distinguishing mature and immature oocytes. Based on these different definitions, it can be concluded that the patients in our study can be categorized as 'relatively poor responders'. In each cycle of this study, at least one MI oocyte was present. The proportion of immature oocytes (GV +MI = 35.7%) in this group of relatively poor responding patients is high compared to the percentages described in the literature after ovarian stimulation in a non-selected group of patients (15–20%; [1,2]) and, as a consequence, the total MI oocyte utilization rate of 76% shows that embryos from in vitro matured MI oocytes were used at high frequency for transfer or cryopreservation.

In the majority of the ICSI cycles, the maturation status of the MI oocytes was checked between 2 to 7 hours after oocyte retrieval. Within this time-frame, 41.3% of the collected MI oocytes extruded their polar body. These maturation rates are comparable with those achieved by others working with MI oocytes retrieved from stimulated cycles. Chian et al. [16] obtained a maturation rate of 46.1% and 52.0% after 6 hrs of in vitro culture and Strassburger et al. [8] obtained 45.1% matured oocytes after 4 hrs of culture. Other studies describe lower maturation rates, like the study of Devos et al. [6] (26.7% maturation after 4 hrs of culture) and the study of Chen et al. [9] (16.4% maturation after 9 hrs of culture). Variations in maturation rates between studies might be explained by different starting and ending points for in vitro maturation. Also the culture conditions, the use of more suitable types of media and/or the addition of serum, growth factors and hormones might influence maturation rates, subsequent fertilization and embryo development of in vitro matured oocytes [16-18]. The group of Balakier et al. [7] performed an exact time recording of polar body extrusion during IVM of MI oocytes. They found that the highest fertilization rate and the lowest incidence of multinucleation were obtained when injection of the oocytes was performed between 3 to 6 hrs after extrusion of the first polar body. This indicates that oocyte maturation is not completed upon reaching the MII stage. In the present study, we did not perform an accurate kinetic experiment, but proper timing of polar body extrusion as well as injection may enhance the outcome of in vitro matured oocytes.

Whatever the conditions of in vitro maturation applied, our study and the majority of other studies show consistently lower fertilization rates of in vitro matured MI oocytes compared to sibling in vivo matured oocytes [6-8]. The proportion of poor quality embryos was higher after in vitro maturation. This is in accordance with other publications, which describe more cleavage arrest [7,8], a higher number of multinucleated blastomeres [7] and a reduced development to the blastocyst stage [9] after IVM. On the contrary, the study of Devos et al. [6], performed on a large group of 896 ICSI cycles, found the same proportions of excellent and fair quality embryos after IVM compared to in vivo matured oocytes. This might be explained by the IVM incubation time of maximum 4 hrs applied in this study. Longer IVM incubation times could result in oocyte ageing. However, we were not able to find a statistical difference in fertilization rate and embryo quality between the different IVM time intervals in the in vitro matured group, although a larger sample would be necessary for a more conclusive statement in the IVM time intervals of >7–11 hrs and 19–26 hrs. Nevertheless, of special interest was the fact that the live birth we obtained from exclusively IVM oocytes originated from an oocyte that was matured in vitro for a period of 22 hrs.

## Conclusion

We may conclude that the use of in vitro matured MI oocytes can be of benefit to obtain pregnancy in patients with a low number of MII oocytes. IVM culture conditions and time schedule for ICSI must be refined to achieve optimum fertilization and development.

## Authors' contributions

LV and JVDE designed the study. LV collected and analyzed the data of the study and wrote the manuscript. All the authors corrected the manuscript and approved the final version. PDS and MD treated the patients.

## References

[B1] Cha KY, Chian RC (1998). Maturation in vitro of immature human oocytes for clinical use. Hum Reprod Update.

[B2] Smitz J, Nogueira D, Vanhoutte L, de Matos DG, Cortvrindt R, Gardner DK, Weissman A, Howles CM, Shoham Z (2004). Oocyte in vitro maturation. Textbook of Assisted Reproductive Technology.

[B3] Stouffer RL, Zelinski-Wooten MB (2004). Overriding follicle selection in controlled ovarian stimulation protocols: quality vs quantity. Reprod Biol Endocrinol.

[B4] Ectors FJ, Vanderzwalmen P, Van Hoeck J, Nijs M, Verhaegen G, Delvigne A, Schoysman R, Leroy F (1997). Relationship of human follicular diameter with oocyte fertilization and development after in-vitro fertilization or intracytoplasmic sperm injection. Hum Reprod.

[B5] Triwitayakorn A, Suwajanakorn S, Pruksananonda K, Sereepapong W, Ahnonkitpanit V (2003). Correlation between human follicular diameter and oocyte outcomes in an ICSI program. J Assist Reprod Genet.

[B6] De Vos A, Van de Velde H, Joris H, Van Steirteghem A (1999). In-vitro matured metaphase-I oocytes have a lower fertilization rate but similar embryo quality as mature metaphase-II oocytes after intracytoplasmic sperm injection. Hum Reprod.

[B7] Balakier H, Sojecki A, Motamedi G, Librach C (2004). Time-dependent capability of human oocytes for activation and pronuclear formation during metaphase II arrest. Hum Reprod.

[B8] Strassburger D, Friedler S, Raziel A, Kasterstein E, Schachter M, Ron-El R (2004). The outcome of ICSI of immature MI oocytes and rescued in vitro matured MII oocytes. Hum Reprod.

[B9] Chen SU, Chen HF, Lien YR, Ho HN, Chang HC, Yang YS (2000). Schedule to inject in vitro matured oocytes may increase pregnancy after intracytoplasmic sperm injection. Arch Androl.

[B10] Dozortsev D, Rybouchkin A, De Sutter P, Qian C, Dhont M (1995). Human oocyte activation following intracytoplasmic sperm injection: the role of the sperm cell. Hum Reprod.

[B11] Ombelet W, De Sutter P, Van der Elst J, Martens G (2005). Multiple gestation and infertility treatment: registration, reflection and reaction – the Belgian project. Hum Reprod Update.

[B12] Fasouliotis SJ, Simon A, Laufer N (2000). Evaluation and treatment of low responders in assisted reproductive technology: a challenge to meet. J Assist Reprod Genet.

[B13] Faber BM, Mayer J, Cox B, Jones D, Toner JP, Oehninger S, Muasher SJ (1998). Cessation of gonadotropin-releasing hormone agonist therapy combined with high-dose gonadotropin stimulation yields favorable pregnancy results in low responders. Fertil Steril.

[B14] De Sutter P, Dhont M (2003). Poor response after hormonal stimulation for in vitro fertilization is not related to ovarian aging. Fertil Steril.

[B15] Moreno C, Ruiz A, Simon C, Pellicer A, Remohi J (1998). Intracytoplasmic sperm injection as a routine indication in low responder patients. Hum Reprod.

[B16] Chian RC, Chung JT, Downey BR, Tan SL. (2002). Maturational and developmental competence of immature oocytes retrieved from bovine ovaries at different phases of folliculogenesis. Reprod Biomed Online.

[B17] Cekleniak NA, Combelles CM, Ganz DA, Fung J, Albertini DF, Racowsky C (2001). A novel system for in vitro maturation of human oocytes. Fertil Steril.

[B18] Roberts R, Franks S, Hardy K (2002). Culture environment modulates maturation and metabolism of human oocytes. Hum Reprod.

